# Lowering Low-Density Lipoprotein Cholesterol Concentration with Plant Stanol Esters to Reduce the Risk of Atherosclerotic Cardiovascular Disease Events at a Population Level: A Critical Discussion

**DOI:** 10.3390/nu12082346

**Published:** 2020-08-06

**Authors:** Helena Gylling, Timo E. Strandberg, Petri T. Kovanen, Piia Simonen

**Affiliations:** 1 Helsinki University Hospital, University of Helsinki, 00014 Helsinki, Finland; timo.strandberg@helsinki.fi; 2 Center for Life-Course Health Research, University of Oulu, 90570 Oulu, Finland; 3 Wihuri Research Institute, 00290 Helsinki, Finland; petri.kovanen@wri.fi; 4 Heart and Lung Center, Cardiology, Helsinki University Hospital, University of Helsinki, 00029, Helsinki, Finland; piia.simonen@hus.fi

**Keywords:** atherosclerosis, cardiovascular disease, cholesterol, lipoprotein, nutrition, plant stanol, risk reduction

## Abstract

Atherosclerotic cardiovascular diseases (ASCVDs) cause every fifth death worldwide. However, it is possible to prevent the progression of ASCVDs by reducing circulating concentrations of low-density lipoprotein cholesterol (LDL-C). Recent large meta-analyses demonstrated that by reducing the dietary intake of saturated fat and cholesterol, it is possible to reduce the risk of ASCVD events. Plant stanols, as fatty-acid esters, were developed as a dietary adjunct to reduce LDL-C levels as part of a heart-healthy diet. They reduce cholesterol absorption so that less cholesterol is transported to the liver, and the expression of LDL receptors is upregulated. Ultimately, LDL-C concentrations are reduced on average by 9–12% by consuming 2–3 g of plant stanol esters per day. In this review, we discuss recent information regarding the prevention of ASCVDs with a focus on dietary means. We also present new estimates on the effect of plant stanol ester consumption on LDL-C levels and the risk of ASCVD events. Plant stanol esters as part of a heart-healthy diet plausibly offer a means to reduce the risk of ASCVD events at a population level. This approach is not only appropriate for subjects with a high risk of ASCVD, but also for subjects at an apparently lower risk to prevent subclinical atherosclerosis.

## 1. Introduction

Cardiovascular diseases (CVDs) are still the most frequent cause of death worldwide [1]. Two-thirds of CVD deaths are caused by atherosclerotic cardiovascular diseases (ASCVDs), such as coronary artery disease (CAD), ischemic stroke, and peripheral artery disease. Thus, ASCVD causes more than one-fifth of all deaths worldwide [1]. The alarming news is that in high-income countries, the declining trend in CVD mortality has ceased and is even increasing, especially among those aged 35–74 years [2]. Serum low-density lipoprotein cholesterol (LDL-C) is the major causal factor underlying ASCVD, and although statins and proprotein convertase subtilisin/kexin type 9 (PCSK9) inhibitors enable effective control of LDL-C in hypercholesterolaemic patients with ASCVD, dietary and other lifestyle measures will remain the backbone of LDL-C control at the population level [3]. Here, we compile and discuss recent information regarding the prevention of ASCVD with a focus on dietary means, especially the intake of dietary fat, cholesterol, and plant stanols, one type of phytosterols. In addition, we present new estimations to predict the effect of plant stanol ester consumption on LDL-C levels and on the risk of ASCVD events. The review is based on our long-term research interest on atherosclerosis, its clinical sequences, and the prevention of ASCVDs including dietary means. Literature database MEDLINE provided the main sources of information.

## 2. Relationship between LDL-C Concentration and ASCVD

### 2.1. Quantification of Outcomes

Compelling evidence from extensive systematic reviews and meta-analyses of pharmacological, dietary, and genetic studies indicates that lowering LDL-C concentrations reduces the risk of ASCVD events, specifically major vascular events (MVEs) denoting fatal or nonfatal CAD, coronary artery revascularization, or stroke [4,5,6,7,8,9,10]. The achieved clinical benefit is dose-dependent and relies on the degree to which LDL-C concentrations are lowered and how low a level of LDL-C is reached. More accurately, based on the results of statin and non-statin trials, such as dietary, ezetimibe, and bile-acid sequestrant interventions, and partial ileal bypass surgery, LDL-C lowering by 1 mmol/L leads to a reduction in the five-year risk of ASCVD events by 21–25% [4,5,6,7,8,9,10]. Thus, up to every fourth atherosclerotic event could be prevented when LDL-C is lowered by 1 mmol/L via upregulation of LDL receptor expression [7].

Based on the results of randomized statin trials, the Cholesterol Treatment Trialists’ (CTT) Collaborators group, an independent group of researchers, demonstrated that the relationship between lowered LDL-C concentrations and the reduced risk of ASCVD events was linear and could be plotted on a regression line [4]. The results of non-statin interventions also confirm this linear relationship. However, genetic studies, in which various gene mutations resulted in lifelong lower LDL-C concentrations compared with control populations, have their own regression line, since 1 mmol/L lower LDL-C levels for decades decreases the risk of CAD by up to 54% [8,9].

LDL-C reductions smaller than 1 mmol/L have an impact on preventing ASCVD events, too. Niemann–Pick C1-like 1 protein (NPC1L1) transports dietary and biliary cholesterol into the enterocytes of the small intestine. Dysfunctional mutations of the *NPC1L1* gene have been found to reduce LDL-C levels and the risk of CAD [11]. The carriers of such mutations had 0.31 mmol/L lower LDL-C concentrations (*p* = 0.04) and a 53% lower risk of CAD (*p* = 0.008) compared with non-carriers. Subsequently, the Improved Reduction of Outcomes: Vytorin Efficacy International Trial (IMPROVE-IT) study demonstrated that ezetimibe, which partly inhibits the function of the NPC1L1 protein and reduces the absorption of cholesterol by about 50% [12], lowered LDL-C levels by 0.43 mmol/L in subjects on a stable statin dose and reduced the risk of ASCVD events by 6.4% (*p* = 0.016) compared with statin alone [13].

### 2.2. Subclinical Atherosclerosis

**Figure 1:**
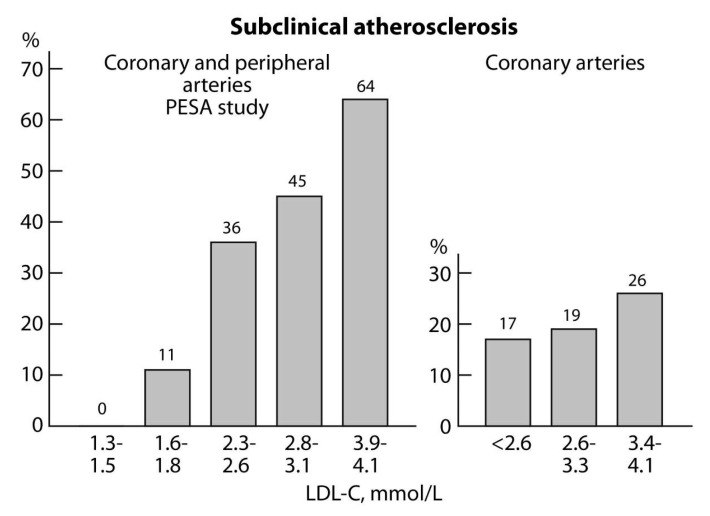
Serum low-density lipoprotein cholesterol (LDL-C) concentrations and the frequency of atherosclerotic changes (calcification or plaques) in coronary and peripheral arteries in subjects without cardiovascular disease risk factors. Modified from [14] Progression of Early Subclinical Atherosclerosis (PESA study), left panel, and [15], right panel.

The concept and evidence of subclinical atherosclerosis in subjects with normal levels of LDL-C and in the absence of other conventional CVD risk factors, such as smoking, hypertension, and elevated fasting plasma glucose or diabetes, also emphasize the necessity and sufficiency of LDL-C in the development of atherosclerosis [14,15]. If LDL-C concentrations alone are markedly increased, such as in the homozygous form of familial hypercholesterolaemia (FH), then atherosclerotic changes are already present in the arterial tree of the patient in early childhood [16]. In studies of two asymptomatic (non-FH) populations with median ages of 45 and 53 years and levels of LDL-C of < 4.1 mmol/L, coronary artery calcification was present in 11% [14] and 21% of the subjects [15], respectively. Peripheral arterial plaques were even more frequent and were present in almost half of the subjects [14]. In both studies, the prevalence of subclinical atherosclerosis significantly increased with increasing LDL-C concentrations (Figure 1). It is remarkable that atherosclerotic plaques were present in 45% of the subjects despite LDL-C concentrations being within the normal reference range (2.8 to 3.1 mmol/L) (Figure 1, left panel). Atherosclerotic plaques appear to start developing once the LDL-C concentration exceeds 1.5 mmol/L, which is considered to be the natural concentration for an adult human [9].

Accordingly, the two essential components to prevent ASCVD events are the concentration of LDL-C and the duration of exposure to elevated levels. In the 2019 European Guidelines for the Management of Dyslipidaemias, the target levels for LDL-C were lower compared with those in earlier guidelines, so that in subjects with high, intermediate, or low ASCVD risks, LDL-C concentrations should be <1.4, <2.6, and <3 mmol/L, respectively [17]. Dietary changes including plant stanol consumption are recommended in the guidelines at all levels of CVD risk as part of lifestyle interventions.

## 3. Dietary Means to Lower LDL-C Concentrations

### 3.1. Dietary Saturated Fat and Cholesterol

At present, circulating concentrations of LDL-C frequently exceed the recommended levels in several populations. At the population level, a heart-healthy diet is recommended as the first step to control hypercholesterolaemia, with the primary focus on reducing the intake of saturated fat and cholesterol [17,18,19], even though there is some debate regarding the role of dietary cholesterol intake [19]. A reduction in the intake of saturated fat from 14.8% to 10.9% of the total energy requirement together with an increase in mono- and polyunsaturated fats can lower levels of LDL-C by 0.31 mmol/L [20]. Silverman et al. [7] estimated that lowering LDL-C by 1 mmol/L by means of established non-statin interventions that work primarily via upregulation of LDL receptor expression (i.e., diet, ezetimibe, bile acid sequestrants, and partial ileal bypass surgery) reduces the risk of ASCVD events by 25%. On this basis, it was estimated, using a surrogate approach, that the LDL-C lowering induced by the above fatty-acid changes [20] would lower the risk of ASCVD events by 7.8% [21].

Dietary cholesterol increases serum total cholesterol and LDL-C concentrations despite several counteractive negative feedback mechanisms [22,23,24,25,26]. In experimental studies, this increase is linear up to a cholesterol intake of 400 mg/day [22]. In population studies, the mean predicted change in LDL-C level for each additional 100 mg of dietary cholesterol/day from 0 to 1500 mg/day was 0.12 mmol/L [27]. Regarding the risk of ASCVD events, each additional intake of 300 mg of dietary cholesterol/day predicted a 17% higher risk of incident CVD and an 18% higher risk of all-cause mortality [28].

### 3.2. Egg Consumption, Serum Cholesterol, and ASCVDs

Consuming half an egg/day (93 mg of cholesterol, on average) predicted a 6% higher risk of incident CVD and an 8% higher risk of all-cause mortality [28]. Thus, eating three to four eggs/week increases the risk of both incident CVD and all-cause mortality. Dietary cholesterol intake has a robust and compelling role in the regulation of LDL-C levels and ASCVD progression. It needs to be acknowledged, however, that conflicting results exist. In a recent study including high- to low-income countries all over the world, with considerably varying dietary habits, no association between egg consumption and the risk of ASCVD events was observed [29]. However, this may be a result of reverse causality and unrecognized confounding factors, because in the stratum of high-income countries, there was a trend toward a similar positive association between egg consumption and the risk of ASCVD events as in the study mentioned above [28].

### 3.3. Plant Stanol Esters as a Dietary Means to Lower LDL-C Concentrations

Phytosterols (plant stanols and sterols) are present in plant-based foods, especially in vegetable oils, cereals, nuts, fruits, and vegetables. The daily intake of plant stanols is low, about 20 mg/day, and ~300 mg/day for plant sterols, about the same as that of cholesterol. It has long been known that dietary phytosterols decrease the absorption of both dietary and biliary cholesterol, and that they lower serum LDL-C concentrations [23,30,31]. Plant stanols are absorbed only slightly, 0.04–0.2%, and less than plant sterols, which are absorbed by 0.5–2% [32]. In the following, we concentrate on plant stanol ester studies.

In 1989, a fat-soluble dietary ingredient, plant stanol esters, was developed [33]. This enabled the preparation of various food products with added plant stanol esters, which could be included in heart-healthy diets to reduce LDL-C concentrations. Consuming 2–3 g/day of plant stanols as fatty-acid esters reduces the absorption of cholesterol by ~41–44% [30,31] by displacing cholesterol from the mixed micelles in the small intestine. Consequently, less cholesterol is transported to the liver so that the hepatic cholesterol pool is diminished, which leads to upregulation of cholesterol synthesis and LDL receptor expression, whereas bile acid metabolism remains unchanged. Ultimately, LDL-C concentrations are reduced on average by 9–12% [34,35,36]. Non-high-density lipoprotein cholesterol (non-HDL-C) concentrations are reduced on average by 10% [37], whereas the levels of high-density lipoprotein cholesterol and serum triglycerides remain generally unchanged [36]. Regarding other potentially beneficial effects for cardiovascular health, plant stanol esters lower plasma plant sterol and oxyphytosterol concentrations from −13% to −38% [38]. They do not influence the circulating lipoprotein a [36] or PCSK9 levels [39] and they decrease the aggregation susceptibility of LDL particles by modifying LDL lipidome [40].

It has been assumed that the nature of cholesterol metabolism has an impact on LDL-C lowering, so that subjects with increased cholesterol synthesis benefit from statin treatment, whereas those with increased cholesterol absorption efficiency benefit from cholesterol absorption inhibition with ezetimibe or with plant stanol esters. There are studies confirming that cholesterol absorption inhibition more effectively lowers LDL-C concentrations in subjects with high than low cholesterol absorption [41,42], but there are also conflicting results [43]. In type 2 diabetes, cholesterol synthesis prevails over cholesterol absorption efficiency, so that in previous studies of diabetics, mean cholesterol absorption efficiency was only 25% [44,45] compared with a population mean of 56% [46]. Despite low cholesterol absorption in these subjects, plant stanol ester consumption of 3 g of plant stanols/day still reduced cholesterol absorption efficiency to 9%, which resulted in LDL-C lowering by 9% and 14%, respectively [44,45].

Because the cholesterol-lowering mechanisms of plant stanol esters differ from those of other cholesterol-lowering dietary components, plant stanol esters and a heart-healthy diet can reduce LDL-C concentrations on average by 1.45 mmol/L (35%) compared with the habitual diet [47]. In addition, plant stanol esters combined with statins have an additive ~10–15% LDL-C- lowering effect depending on the daily plant stanol dose [36]. In 2009, The Commission of the European Community accepted a health claim for plant stanol esters, “Plant stanol ester consumption lowers blood cholesterol concentration. High cholesterol level is a risk factor for coronary artery disease”. The European Food Safety Authority (EFSA) later performed a scientific evaluation, and based on the results of this evaluation, concluded that plant stanol ester consumption equal to 3 g of plant stanols/day lowers LDL-C concentrations on average by 11.4% (95% confidence interval 9.8–13.0) [35].

The efficacy and safety of plant stanol ester consumption was evaluated in clinical randomized controlled interventions and in in vitro and in vivo studies in internationally accredited research laboratories [34,36]. A meta-analysis of clinical interventions was published in 2011, covering 62 plant stanol interventions, of which 57, i.e., most of them, were plant stanol ester trials [34]. The studies demonstrated that consumption of plant stanol esters equal to 2 and 3 g of plant stanols/day lowered LDL-C concentrations on average by 9% and 11.8%, respectively. Food safety authorities in both Europe and the USA, and the FAO/WHO have accepted the long-term use of plant stanol esters as an effective and safe means to lower LDL-C concentrations [35,48,49,50]. In addition, plant stanols are included in the recommendations of the 2019 European Guidelines for the Management of Dyslipidaemias [17]. Accordingly, in hypercholesterolaemic subjects also including diabetic and obese subjects, the recommended plant stanol ester dose is 2–3 g plant stanols/day. Since the subclinical atherosclerosis is frequent even in subjects with LDL-C levels < 3 mmol/L [14,15], plant stanol ester consumption equal to 2–3 g plant stanols/day effectively lowers LDL-C concentration also in subjects with baseline LDL-C levels < 3 mmol/L [51] to prevent subclinical atherosclerosis.

### 3.4. Plant Stanol Esters and Egg Consumption

Regarding the observation that additional intake of half an egg/day had an impact on ASCVD events [28], we were interested in estimating the degree to which plant stanol ester consumption could diminish the absorption of cholesterol from one additional egg/day. We based our studies on data from an earlier randomized, controlled trial concerning plant stanol consumption of 2.7 g/day as esters in 22 subjects [30]. Dietary cholesterol intake was determined from seven-day food diaries, and the amount of intestinal biliary, dietary, and total cholesterol fluxes, and cholesterol absorption, was calculated on the basis of analyses of fecal samples from three-day fecal collections. The diets included the home diet with and without plant stanol esters. We estimated the metabolic consequences, approximating that one egg (per day) containing 200 mg of cholesterol [52] was added to these two diets (Table 1).

In the original study above [30], estimating the addition of plant stanol esters to the home diet reduced the amount of absorbed cholesterol by almost half, i.e., by 212 mg/day (*p* < 0.05; Table 1). In our surrogate model, adding one medium-sized egg/day to the home diet without plant stanol esters increases the amount of absorbed cholesterol by 81 mg/day. When plant stanol ester consumption was added to the egg-enriched home diet, we estimated cholesterol absorption efficiency to be reduced by 43%. Accordingly, plant stanol ester consumption could practically prevent the increase in absorbed cholesterol from the additional one egg/day. Thus, dietary plant stanols enable people to benefit from the nutritionally good composition of eggs without the concern of increased cholesterol intake. It is noteworthy that the 2019 European Guidelines for the Management of Dyslipidaemias [17] and the recent science advisory from the American Heart Association [19] both advise subjects at an elevated risk of ASCVD to control their cholesterol intake.

### 3.5. Plant Stanol Esters and the Risk of ASCVD Events

**Figure 2:**
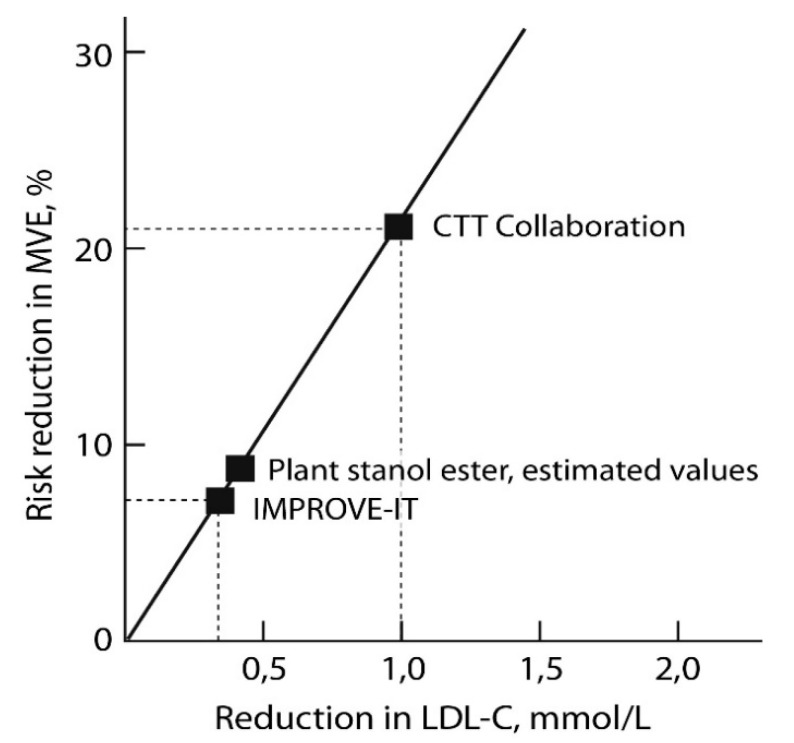
Estimated clinical benefit and reduction of low-density lipoprotein cholesterol (LDL-C) concentrations by plant stanol ester consumption (3 g plant stanols/day) depicted on the regression line published by the CTT Collaboration, and on which the IMPROVE-IT study results were plotted. The estimations are based on data presented in [4,34], and the figure is modified from data in [4,13]. CTT Collaboration = Cholesterol Treatment Trialists’ (CTT) Collaborators [4], IMPROVE-IT = Improved Reduction of Outcomes: Vytorin Efficacy International Trial [13]. MVE = Major Vascular Events; fatal or nonfatal coronary artery disease, coronary artery revascularization, or stroke [4].

Plant stanol ester consumption lowers LDL-C concentrations, but no ASCVD endpoint studies are available. According to the expert panel of the European Atherosclerosis Society on phytosterols, an ASCVD endpoint study is impossible to undertake [36]. Such an intervention would require a very large number of subjects (>50,000) for adequate power, and a very long duration [36]. Recently, results concerning the effects of ezetimibe in reducing the risk of ASCVD events in the IMPROVE-IT study were matched to the CTT Collaborators’ regression line (Figure 2).

Silverman et al. [7] later established that LDL-C lowering by non-statin interventions that work primarily via upregulation of LDL receptor expression, e.g., ezetimibe treatment, reduces the risk of ASCVD events to a similar degree as statin treatment. Ezetimibe and plant stanol esters have similarities in terms of their cholesterol-lowering mechanisms, since both result in reduced cholesterol absorption and upregulated LDL receptor expression [13,53]. For this reason, we were interested in determining whether the regression equation presented by the CTT Collaborators could be used to estimate the risk of ASCVD events in relation to plant stanol ester consumption.

Based on the results of the plant stanol meta-analysis [34], we calculated the changes in LDL-C concentrations with different daily intakes of plant stanols using the weighted analysis method (Table 2). The changes in the risk of ASCVD events were estimated using the CTT Collaborators’ regression equation [4]. The novel estimations demonstrated that plant stanol consumption of 2 g/d reduced LDL-C concentrations by 0.33 mmol/L, which was expected to reduce the risk of ASCVD events by 6.9%. For plant stanol consumption of 3 g/day, the respective estimates were −0.42 mmol/L and –8.8% (Figure 2). Since it is impossible to study the actual clinical outcomes, using the above surrogate outcome (LDL-C reduction) approach can be justified. The similarity of the LDL-C- lowering mechanisms of ezetimibe and plant stanol esters supports the validity of such surrogate estimates, at least in this particular case.

## 4. Conclusions

The reduction of LDL-C concentrations is the primary target to reduce the risk of ASCVD events. By reducing the number of circulating LDL particles, which form the majority of the circulating atherogenic lipoproteins, it is possible to prevent ASCVD events. However, the rate of success depends on how early the LDL-C concentration is reduced and the degree to which low levels are achieved. Based on the main findings by Brunner et al. [54], increased LDL-C and other non-HDL-C concentrations in middle age are strongly associated with a long-term risk of ASCVD.

The results of recent large meta-analyses once again highlight the remarkable role that dietary habits (especially high-level saturated fat and cholesterol intake) possess in the regulation of cholesterol metabolism and LDL-C concentrations. They also highlight that by reducing the intake of both saturated fat and cholesterol, it is possible to successfully reduce LDL-C levels and the risk of ASCVD events. Plant stanol esters were developed as a dietary adjunct to lower LDL-C concentrations as part of a heart-healthy diet. They reduce cholesterol absorption, and it can be estimated, on the basis of the results of a previously published study [30], that plant stanol ester consumption could prevent the additional absorption of cholesterol derived from one egg/day.

Unfortunately, it is not possible to carry out ASCVD endpoint studies in connection with plant stanol ester foods because of the large number of subjects needed and the challenge of controlling diets for a very long period. Appropriate use of a surrogate end point requires in-depth understanding of the multiple causal pathways of the disease process in question and the intended as well as unintended mechanisms of action of treatment intervention [55]. Hence, we think that the requirements for use of a surrogate endpoint (LDL-C concentrations) are fulfilled in the cascade of plant stanol ester → LDL-C → ASCVD, and we therefore consider that a surrogate outcome approach based on published scientific evidence is justified [55]. This approach provided a quantitative estimate concerning lowering of LDL-C concentrations achieved through intake of plant stanol esters and a corresponding reduction in ASCVD events.

In summary, plant stanols, as fatty-acid esters used as part of a heart-healthy diet, markedly lower LDL-C concentrations and plausibly offer a feasible means at a population level to reduce the risk of ASCVD events. This approach is not only appropriate for subjects with an established high risk of ASCVD, but also to prevent subclinical atherosclerosis in lower-risk subjects.

## Figures and Tables

**Table 1:** Estimated daily amounts of biliary, dietary, and total cholesterol flux in the small intestine, and daily absorbed cholesterol during intake of different diets.

Diet	Biliary Cholesterol Flux, (mg/day)	Dietary Cholesterol Flux, (mg/day)	Total Cholesterol Flux, (mg/day)	Absorbed Total Cholesterol, (mg/day)	Difference Versus Home Diet (mg/day)
Home diet (data from original study [30])	971	247	1218	497	
Home diet + 2.7 g plant stanols/day (data from original study [30])	971	247	1218	285	–212
Home diet + 1 egg (200 mg cholesterol) *	971	447	1418	578	+ 81
Home diet + 1 egg (200 mg cholesterol) + 2.7 g plant stanols/day *	971	447	1418	332	–165

* Calculations based on data presented in [30]: study population: 22 women, mean age 51 years and mean body weight 68 kg. Cholesterol absorption efficiency was 40.8% during home diet and 23.4% during the plant stanol ester diet. Total cholesterol flux = fecal cholesterol/(1–cholesterol absorption efficiency), biliary cholesterol flux = total cholesterol flux—dietary cholesterol.

**Table 2:** Change in LDL-C concentrations by plant stanol ester consumption and its predicted impact on the risk of ASCVD events.

Plant Stanol Consumption, g/day	Change in LDL-C, mmol/L	Change in LDL-C (%)	Change in ASCVD Events (%)
1	–0.19	–5.4	–4.0
1.5	–0.27	–7.4	–5.7
2	–0.33	–9.2	–6.9
3	–0.42	–11.8	–8.8
4	–0.48	–13.7	–10.1

LDL-C = low-density lipoprotein cholesterol; ASCVD = atherosclerotic cardiovascular disease; ASCVD events = major vascular events (fatal or nonfatal coronary artery disease, coronary artery revascularization, or stroke [4]. Regarding plant stanol ester dose and LDL-C concentration, the calculations are based on the results of a large meta-analysis of clinical randomized, controlled plant stanol ester studies [34], Table 5 ‘Stanol ester, weighted analysis, no dose restriction’. Calculations concerning ASCVD events were based on data in [4].
